# Rapid detection of *Pseudomonas aeruginosa *from positive blood cultures by quantitative PCR

**DOI:** 10.1186/1476-0711-9-21

**Published:** 2010-08-04

**Authors:** Vincent Cattoir, Audrey Gilibert, Jeanne-Marie Le Glaunec, Nathalie Launay, Lilia Bait-Mérabet, Patrick Legrand

**Affiliations:** 1 Laboratoire de Bactériologie-Virologie-Hygiène, Hôpital Henri Mondor, Assistance Publique-Hôpitaux de Paris, Créteil, France; 2 Laboratoire de Microbiologie, CHU Côte de Nacre, Caen, France

## Abstract

**Background:**

*Pseudomonas aeruginosa *is responsible for numerous bloodstream infections associated with severe adverse outcomes in case of inappropriate initial antimicrobial therapy. The present study was aimed to develop a novel quantitative PCR (qPCR) assay, using *ecfX *as the specific target gene, for the rapid and accurate identification of *P. aeruginosa *from positive blood cultures (BCs).

**Methods:**

Over the period August 2008 to June 2009, 100 BC bottles positive for gram-negative bacilli were tested in order to evaluate performances of the qPCR technique with conventional methods as gold standard (i.e. culture and phenotypic identification).

**Results:**

Thirty-three strains of *P. aeruginosa*, 53 strains of Enterobactericaeae, nine strains of *Stenotrophomonas maltophilia *and two other gram-negative species were isolated while 3 BCs were polymicrobial including one mixture containing *P. aeruginosa*. All *P. aeruginosa *clinical isolates were detected by qPCR except a single strain in mixed culture. Performances of the qPCR technique were: specificity, 100%; positive predictive value, 100%; negative predictive value, 98.5%; and sensitivity, 97%.

**Conclusions:**

This reliable technique may offer a rapid (<1.5 h) tool that would help clinicians to initiate an appropriate treatment earlier. Further investigations are needed to assess the clinical benefit of this novel strategy as compared to phenotypic methods.

## Background

*Pseudomonas aeruginosa *is a major human opportunistic pathogen responsible for numerous nosocomial infections, especially within intensive care units [1]. In US hospital settings, *P. aeruginosa *is the seventh (i.e. 4.3%) most frequently isolated pathogen from the bloodstream, with a crude mortality exceeding 25% [2]. Although rarely responsible for community-acquired infections, *P. aeruginosa *represents 6.8% of bacteremia caused by gram-negative rods [3]. Since *P. aeruginosa *bacteremia is clinically indistinguishable from other gram-negative bacterial bloodstream infections, it has been demonstrated that an inappropriate initial antimicrobial therapy was associated with severe adverse outcomes [4-6].

Because standard phenotypic methods are time consuming and most have inherent limitations, molecular techniques have shown to be a rapid and reliable approach for the identification of bacterial pathogens [7]. Therefore, several PCR-based methods have already been described to identify *P. aeruginosa*, especially in respiratory samples from cystic fibrosis patients. Different molecular targets have been employed such as 16 S rRNA, *algD*, *oprI*, *oprL*, *toxA*, *gyrB*, and *ecfX *genes [8-13]. Since false-positive results (with 16 S rRNA and *oprI *genes) as well as false-negative results (with *algD *and *toxA *genes) have been reported, the *ecfX *gene is a suitable target for species-specific identification of *P. aeruginosa *isolates [11,13].

While identification of *P. aeruginosa *from a positive blood culture (BC) takes at least 24 h using conventional techniques, molecular identification directly from positive BCs could be an interesting alternative leading to a rapid and accurate species-level identification with subsequent adequate empirical treatment. However, PCR detection of *P. aeruginosa *from positive BCs has been poorly investigated [14,15], and the authors used *oprI *and *oprL *as target genes, described previously as non-100% specific [8,11].

In this study, we have developed a novel quantitative PCR (qPCR) assay, using *ecfX *as the specific target gene, for the rapid and accurate identification of *P. aeruginosa *from positive BCs

## Methods

### Clinical specimens and phenotypic identification

From August 2008 to June 2009, a total of 100 positive BCs from 100 inpatients were included. For each patient, a pair of aerobic (BacT/ALERT FA) and anaerobic (BacT/ALERT FN) bottles was taken, then incubated in the BacT/ALERT automated continuous monitoring system (bioMérieux, Marcy-l'Etoile, France). The distribution of positive BCs by bottle type was as follows: 87 aerobic (87%) and 13 (13%) anaerobic. All these BCs showed gram-negative rods at the direct examination, and most of them presented a Gram-staining and/or a motility compatible with those of the species *P. aeruginosa*. Positive bottles were inoculated aerobically and anaerobically at 37°C for 24-48 h onto trypticase-soy agar, Drigalski agar, 5% horse blood agar and chocolate agar plates, and the concentration of bacteria was determined by quantitative culture (10-fold serial dilutions from 10^-1 ^to 10^-10^). Strains were identified by colony morphology, oxidase reaction, and results of the API 20 E system (bioMérieux).

### DNA extraction

From a 0.5-ml aliquot of blood, template DNA was prepared by using a simple and rapid boiling procedure, taking less than 20 min [16,17]. Briefly, the aliquot was centrifuged at 850 × g for 2 min to remove the charcoal. The supernatant was centrifuged at 11,500 × g for 5 min. The resulting pellet was resuspended in 200 μl of a lysis buffer containing 1% Triton X-100, 0.5% Tween 20, 10 mM Tris-Hcl (pH 8.0), and 1 mM EDTA and incubated at 100°C for 10 min. After centrifugation for 2 min at 850 × g, the supernatant was directly used for PCR.

### Quantitative PCR

The qPCR assay was performed with the LightCycler v.2.0 instrument (Roche, Meylan, France). Oligonucleotide primers and fluorescent-labeled hybridization probes were designed for amplification and sequence-specific detection of a 152-bp fragment within the *ecfX *gene (Table 1). The amplification mixture consisted of 2 μl of 10× LightCycler FastStart DNA Master Hybridization Probes mixture (Roche), 2 mM MgCl_2_, 0.5 μM each primer, 0.2 μM each probe, and 5 μl of template DNA in a final volume of 20 μl. A suspension of Tris-EDTA and a DNA extract of *P. aeruginosa *ATCC 27853 were used as negative and positive controls, respectively. Following an initial denaturation at 95°C for 10 min, the 45-cycle amplification profile consisted of heating a 95°C segment for 10 s, a 50°C segment for 10 s, and a 72°C segment for 20 s. The presence of amplified DNA was measured by detection of emitted fluorescence at 705 nm, and the final result was available in ~1.5 h. In parallel, PCR inhibition control was performed for all samples in the same run by using a second reaction tube containing 100 ng of DNA extract from the positive control.

**Table 1:** Oligonucleotide primers and LightCycler hybrizidation probes used in the PCR assay

Oligonucleotides	Sequence (5'-3')	Target gene	Nucleotide position
*ecfX*-F	TTCCATGGCGAGTTGCT	*ecfX*^b^	46-62
*ecfX*-R	CGGGCGATCTGGAAAAGAA		179-197
*ecfX*-HP-1	GCTGAAATGGCCGGGCC-[FAM]		135-151
*ecfX*-HP-2	[LC705]-CAATCGGTCGAGCAGCCGC-Ph		154-172

^a ^[FAM], fluorescein; [LC705], LightCycler™-Red 705; Ph, 3'-phosphate. ^b ^Extracytoplasmic function gene (GenBank accession no. DQ996551).

### Limit of detection

A plasmid standard curve was constructed by ligating the 152-bp PCR product from *P. aeruginosa *ATCC 27853 into the commercial plasmid vector, pCR2.1-TOPO plasmid vector from the TOPO TA Cloning Kit (Invitrogen, Cergy-Pontoise, France) following the manufacturer's recommendations to produce plasmid pRT-*ecfX*. This plasmid was transformed into competent TOP10 *Escherichia coli *cells (Invitrogen), and transformants were selected on media containing 30 μg/mL kanamycin. Plasmid DNA was extracted and purified from one transformant using the HiSpeed Midi Kit (Qiagen, Valencia, CA) according to the manufacturer's instructions, resuspended in elution buffer (Qiagen) and sequenced to determine the presence of the *ecfX *insert and its orientation. Plasmid DNA concentration was determined by using the NanoDrop ND-1000 spectrophotometer (Thermo Scientific, Illkirch, France), and the plasmid DNA reference material was then serially diluted to obtain 10 to 10^10 ^plasmid genome equivalents for standard curve analysis. Quantitative analysis was carried out with the LightCycler software version 3.5 (Roche). The ratio of signals measured at 705 nm/signals measured at 530 nm was used to calculate the crossing point values.

## Results

**Figure 1:**
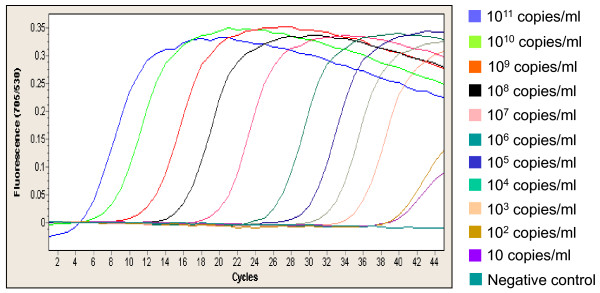
**qPCR amplification curves of plasmid DNA reference material with 11 external DNA concentrations (from 10^11 ^to 10 copies/ml)**.

**Figure 2:**
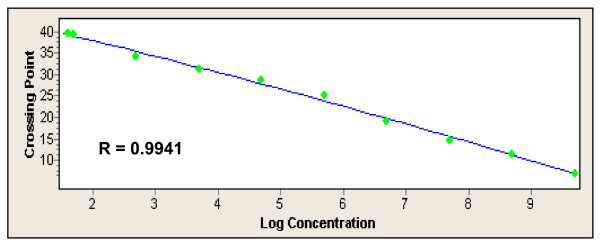
**Calibration curve of qPCR using serial dilutions of plasmid DNA reference material (see Figure 1)**.

Culture of the 100 BCs isolated 33 strains of *P. aeruginosa *(including 2 recovered in anaerobic bottles), 53 strains of Enterobacteriaceae (including 22 *Escherchia coli*), 9 strains of *Stenotrophomonas maltophilia*, 2 other gram-negative species, and 3 BCs were polymicrobial of which one mixture of *Klebsiella pneumoniae *and *P. aeruginosa *(Table 2). Note that the presence of PCR inhibition was observed only for two (2%) DNA preparations. Out of the 95 monomicrobial cultures, the real-time *ecfX *PCR assay was 100% sensitive and 100% specific for detecting *P. aeruginosa*. However, the PCR assay could not detect the presence of *P. aeruginosa *in a single polymicrobial culture with *K. pneumoniae*. By performing the PCR assay directly from the *P. aeruginosa *isolate, we could amplify *ecfX*, demonstrating that this problem was not due to an absence or a sequence variation of the gene. By quantitative culture, we found that the concentration of *P. aeruginosa *in this BC was very low (20 CFU/ml) as compared with that of *K. pneumoniae *(10^8 ^CFU/ml). Limit of detection was estimated at 10^2 ^CFU/ml (Fig. 1 and Fig. 2) leading that the absence of signal by PCR could be explained by a default of sensitivity in this peculiar case. Taken into account the 98 BCs with interpretable results (98%), the qPCR assay showed 100% of specificity and PPV, 98.5% of NPV, and 97% of sensitivity.

**Table 2:** *ecfX *qPCR testing of *P. aeruginosa *from 98 positive blood culture bottles

Organisms	No. of samples detected		Total no. of samples
		
	**PCR**^**+**^	**PCR**^**-**^	
***Pseudomonas aeruginosa***	**33**	**0**	**33**
*Escherichia coli*	0	21	21
*Stenotrophomonas maltophilia*	0	9	9
*Klebsiella pneumoniae*	0	8	8
*Enterobacter cloacae*	0	7	7
*Citrobacter koseri*	0	4	4
*Enterobacter aerogenes*	0	3	3
*Proteus mirabilis*	0	3	3
*Morganella morganii*	0	3	3
*Serratia marcescens*	0	1	1
*Pantoea *spp.	0	1	1
*Brevundimonas diminuta*	0	1	1
*Bacteroides fragilis*	0	1	1
*E. coli *+ *Klebsiella oxytoca*^a^	0	1	1
*E. cloacae *+ *K. oxytoca*^a^	0	1	1
*K. pneumoniae *+ ***P. aeruginosa***^a, b^	**0**	**1**	**1**

^a ^Polymicrobial blood cultures. ^b^, 20 CFU/ml of *P. aeruginosa *and 10^8 ^CFU/ml of *K. pneumoniae *were recovered by quantitative culture.

## Discussion

Even if the motility in an aerobic bottle may be a good predictive value of *P. aeruginosa *isolation, the observation of non-motile rods or atypical motility is regularly observed [18] as well as the growth of this aerobe in anaerobic bottles (6% in our study) [19]. By comparison with the new FDA-approved technique (i.e. fluorescence in situ hybrizidation with peptide nucleic acid probes) directly used on positive BCs, this novel qPCR method showed similar performances but appeared faster (1.5 h vs 2.5 h) [20,21]. Nevertheless, a limit of the qPCR technique is the potential presence of PCR inhibitors (e.g. charcoal, haemoglobin) in BC specimens.

The timely and accurate information provided by this qPCR assay would help clinicians identify *P. aeruginosa *bacteremia, and thereby initiate adequate antimicrobial therapy 18 to 24 h earlier than would be possible with conventional methods, as previously described for *Staphylococcus aureus *[16,17] and *K. pneumoniae *[22]. However, as opposed to *S. aureus *and methicillin resistance, it is difficult to predict an useful antibiotic susceptibility profile of *P. aeruginosa *by molecular techniques even if it is possible to detect several resistance genes such as emerging carbapenemase genes [7]. Indeed, the choice of antibiotic treatment has to consider national and local epidemiology since multidrug resistant *P. aeruginosa *isolates are increasingly reported worldwide [23]. Therefore, in order to minimize the risk of inappropriate treatment, the use of combination antimicrobial therapy, until susceptibility results become known, may be preferable in certain situations [5,6].

## Conclusions

As a conclusion, we developed a promising qPCR technique that offers a fast (<1.5 h) tool with high sensitivity and specificity for the identification of *P. aeruginosa *from positive BCs. Further investigations will be perform to evaluate the clinical impact of this novel strategy as compared to conventional methods.

## Competing interests

The authors declare that they have no competing interests.

## Authors' contributions

Each author acknowledges that he has contributed in a substantial way to the work described in the manuscript and its preparation. All authors have read and approved the final manuscript.
